# Slow-Release H_2_S Donor Anethole Dithiolethione Protects Liver From Lipotoxicity by Improving Fatty Acid Metabolism

**DOI:** 10.3389/fphar.2020.549377

**Published:** 2020-09-23

**Authors:** Chengcheng Zhao, Nannan Yu, Wenqun Li, Hualin Cai, Mouze Liu, Yanjie Hu, Yiping Liu, Mimi Tang

**Affiliations:** ^1^ Department of Pharmacy, The Second Xiangya Hospital, Central South University, Changsha, China; ^2^ Institute of Clinical Pharmacy, Central South University, Changsha, China; ^3^ Department of Stomatology, Suiyang County People’s Hospital, Zunyi, China; ^4^ Department of Pharmacy, Xiangya Hospital, Central South University, Changsha, China; ^5^ Institute for Rational and Safe Medication Practices, National Clinical Research Center for Geriatric Disorders, Xiangya Hospital, Central South University, Changsha, China

**Keywords:** hydrogen sulfide, lipotoxicity, liver injuries, palmitic acid, oleic acid, fatty acid β-oxidation

## Abstract

“Lipotoxicity” induced by free fatty acids (FAs) plays a central role in the pathogenesis of many metabolic diseases, with few treatment options available today. Hydrogen sulfide (H_2_S), a novel gaseous signaling molecule, has been reported to have a variety of pharmacological properties, but its effect on FAs metabolism remains unclear. The purpose of this study was to investigate the effect and mechanisms of anethole dithiolethione (ADT, a sustained-release H_2_S donor) on hepatic FAs metabolism. ADT was administered daily for 4 weeks in male Syrian golden hamsters fed a high fat diet (HFD), and FAs profiles of liver tissues were analyzed using GC-MS. The results showed that in HFD-fed hamsters, ADT treatment significantly reduced the accumulation of toxic saturated and monounsaturated fatty acids (C16:0, C18:0, C16:1, and C18:1n9), while increased the content of n-6 and n-3 series polyunsaturated fatty acids (C20:3n6, C20:4n6, and C22:6n3). Mechanistically, ADT obviously inhibited the overexpression of acetyl-CoA carboxylase1 (ACC1), fatty acid synthase (FAS), and stearoyl-CoA desaturase1 (SCD1), and up-regulated the levels of fatty acid transport proteins (FATPs), liver fatty acid binding protein (L-FABP), carnitine palmitoyltransferase 1α (CPT1α), fatty acid desaturase (FADS)1 and FADS2. Notably, ADT administration significantly promoted Mitofusin1-mediated mitochondrial fusion and fatty acid β-oxidation. These findings suggest that ADT plays a beneficial role by regulating the synthesis, desaturation, β-oxidation, uptake, binding/isolation, and transport of FAs. In conclusion, ADT is effective in improving FAs metabolic disorders and liver injuries caused by HFD, which renders ADT a candidate drug for lipotoxicity-induced diseases.

## Introduction

Fatty acids (FAs) are indispensable sources of energy in cells, and also important bioactive mediators involved in many homeostasis processes, including metabolism and regulating inflammatory immune responses (Masoodi et al., 2015). However, unlike adipocytes, which possess a strong capacity to store excessive free fatty acids (FFAs) in lipid droplets in the form of triglycerides, lipid overload in non-adipose tissues (such as heart, liver, kidney, skeletal muscle, and pancreatic β-cells) causes lipotoxicity, leading to cell dysfunction or death. Lipotoxicity has been reported to be mainly caused by long-chain saturated fatty acids (SFAs), especially palmitic acid (PA, C16: 0) (Leamy et al., 2013). Accumulating data suggest that abnormal FAs metabolism and its induced lipotoxicity are closely related to the risk of developing non-alcoholic fatty liver disease (NAFLD), diabetes, atherosclerosis, heart failure, and even multiple cancers (Afonso et al., 2016; Blucher and Stadler, 2017; Cansancao et al., 2018; Jiang et al., 2019; Zhou et al., 2019). Therefore, inhibition of FAs-related lipotoxicity represents a potential therapeutic strategy, which is of great interest.

The liver is the main metabolic organ of FAs, and the imbalance in its synthesis, uptake and disposal (mainly including mitochondrial oxidation and endoplasmic reticulum re-esterification) will induce lipotoxicity, further leading to the body dysfunction. Mitochondrial β-oxidation, the most important metabolic pathway of FAs, is mainly regulated by rate-limiting enzymes such as carnitine palmitoyl-transferase 1α (CPT1α), which serves as a gatekeeper for FAs to enter mitochondria. In addition, it has been demonstrated that in starved mitochondrial fusion protein 1 knockout (Mitofusin1KO) mouse embryonic fibroblasts (MEFs), mitochondria were fragmented, reducing the β-oxidation rate and leading to lipid accumulation in lipid droplets (Rambold et al., 2015; Wrighton, 2015). However, there are no published data on the relationship between liver FAs metabolism and Mitofusin1 expression during nutrient oversupply.

Hydrogen sulfide (H_2_S), a well-known novel gaseous signaling molecule, is increasingly recognized as a crucial regulator of cardiovascular diseases. H_2_S in mammalian tissues is synthesized endogenously by three enzymes: cystathionine-β-synthase (CBS), cystathionine-γ-lyase (CSE) and 3-mercaptopyruvate sulfurtransferase (3-MPST). Emerging data indicate that endogenous H_2_S level is significantly negatively correlated with abnormal lipid metabolism, including hyperlipidemia, NAFLD, and atherosclerosis(Mani et al., 2013; Sun et al., 2015; Li et al., 2017). Exogenous H_2_S donors (NaHS, GYY4137, etc.) can significantly improve these metabolic diseases through multiple properties, such as anti-inflammatory, antioxidant, inhibiting foam cell formation, improving endothelial function, and activating liver autophagy (Liu et al., 2013; Sun et al., 2015; Durante, 2016; Li et al., 2017). And more notably, a recent study found that FFAs up-regulate the liver expression of 3-MPST, and subsequently inhibit the CSE/H_2_S pathway, impairing the endogenous synthesis of H_2_S, and leading to NAFLD (Li et al., 2017). However, the effect of H_2_S on FAs metabolism in the liver remains unclear.

**Figure 1:**
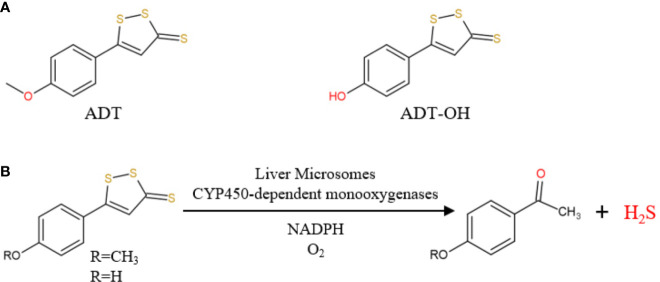
**(A)** Chemical structure of ADT and ADT-OH; **(B)** ADT and ADT-OH are metabolized by hepatic microsomes to form H_2_S (from Dulac et al., 2019).

At present, H_2_S-releasing “drugs” used in research have been largely limited to simple sulfide salts, mainly NaHS. However, NaHS, the so-called immediate-release H_2_S donor, releases excessive H_2_S instantaneously and therefore does not mimic the production of endogenous H_2_S (Li et al., 2008). Anethole dithiolethione (ADT; 5-(4-methoxyphenyl)-3H-1,2-dithiole-3-thione), clinically used as a hepatoprotective and choleretic drug, is also a prodrug of H_2_S (Wang et al., 2014; Szabo and Papapetropoulos, 2017). In vivo, ADT is rapidly metabolized into the active metabolite ADT-OH, with a half-life of about 3.1 and 4.4 h, respectively(Li W. et al., 2008; Wang et al., 2014). Compared with NaHS, ADT releases H_2_S and significantly increases serum H_2_S levels over a long period of time. In 2019, Dulac et al. proposed the first detailed mechanism for the production of H_2_S by ADT and ADT-OH in the presence of rat liver microsomes, NADPH and O_2_ (**Figure 1**) (Dulac et al., 2019). Therefore, this article aims to investigates how clinically available sustained-release H_2_S donor ADT affects hepatic fatty acid metabolism under high-fat diet (HFD) and explores its possible mechanisms.

## Materials and Methods

### Drug

ADT (Lot.N1017A) was purchased from Dalian Meilun Biotechnology Co., LTD., and its clinical dosage is 75 mg per day. The dosage of ADT in Syrian golden hamsters was calculated by human equivalent dose (HED) based on body surface area (Huang et al., 2015), with a conversion dose of 9.25 mg/kg. Therefore, the dose gradient of ADT in this study was set as: 5, 10, 20, and 40 mg/kg. ADT is insoluble in water, suspended in 0.5% sodium carboxymethylcellulose (CMC-Na) and 0.5% soybean lecithin, administered daily by gavage.

### Animals and Diets

This study was reviewed and approved by the Laboratory Animal Care and Welfare Committee of Central South University (Approval No. 2018sydw0215). Fifty-four male Syrian golden hamsters (80–100 g) were purchased from Beijing Vital River Laboratory Animal Technology Co., Ltd. (Qualified Certificate No. SCXK Jing 2016-0011). Hamsters were housed in IVC cages and free access to diet and water, which subjected to a 12-h light/dark cycle with a relative humidity of 50% ± 10% and a temperature of 22°C to 25°C.

Trophic Animal Feed High-Tech Co. Ltd provided high-fat diets and control diets, both of which are formulated based on purified ingredients. The control diet was designed to meet all of the hamster’s nutritional requirements. The cholesterol and lard in the high-fat diet were 2/1000 g and 117/1000 g, respectively (**Table 1**).

**Table 1:** The composition of the high-fat and control diets used in this study.

Compositions	Content	
	High-fat diet	Control diet
Casein	201	171
Corn starch	323	475
Dextrin	0	127
Sucrose	201	96
Soybean oil	47	40
Lard	117	0
Cellulose	50	42
Minerals	41	35
Vitamins	11	10
Methionine	3	3
Choline chloride	2	2
Cholesterol	2	0
TBHQ	0.033	0.008
Total	1000	1000

### Experimental Design

After adaptation for one week, 54 hamsters were randomly divided into six groups, with 9 animals in each group. (1) control group: control diet with vehicle (0.5% CMC-Na and 0.5% soybean lecithin) treatment; (2) HFD group: high-fat diet with vehicle (0.5% CMC-Na and 0.5% soybean lecithin) treatment; (3–6) HFD+ADT group: high-fat diet with different doses of ADT. ADT and/or vehicle were administered to the hamster by gavage once a day for 4 weeks. After 4 weeks, all hamsters fasted overnight and were euthanatized at the end of the experiment. Blood samples were collected from the heart and kept at room temperature for 1 h before centrifugation at 3500 rpm for 10 min. The serum was separated and stored at −80°C until analysis. The left lobe of liver was fixed in 4% paraformaldehyde solution, and the rest was stored at −80°C after quick-freezing by liquid nitrogen.

### Biochemical and Histological Analysis

Serum glutamate alanine aminotransferase (ALT), aspartate aminotransferase (AST) and total bile acid (TBA) were measured by automatic biochemical analyzer (Hitachi 7600-210) in the laboratory of Second Xiangya Hospital. The fixed liver tissue was embedded in paraffin blocks, and 4-μm-thick slices were cut and stained with hematoxylin and eosin (HE) to observe histopathological changes.

### Sample Pretreatment and FFAs Determination

FFAs in liver tissue were extracted and measured by GC-MS (Agilent 7890 A/5975 C) according to the method described by Tang et al. (2018). In short, 750 μl of dichloromethane and methanol (CH_2_Cl_2_: CH_3_OH = 1:2) mixture was added to liver tissue and homogenized, followed by centrifugation to obtain the supernatant. Then, 100 μM butylated hydroxytoluene (BHT, to prevent lipid peroxidation), 250 μl CH_2_Cl_2_ and 250 μl water were sequentially added to the supernatant and vortexed for 30 s. We transferred the lower phase, centrifuged again to take the supernatant which then evaporated to dryness under nitrogen. The residue was dissolved in n-hexane, 10 μl internal standard (heptadecanoic acid) and 2 ml 0.5 M KOH-MeOH were added, followed by water bath heating at 60°C for 20 min. After cooling for 10 min, 3 ml of 12.5% H_2_SO_4_ in methanol solution (to methylate the sample) was added and heated again at 60°C in the water bath for 1 h. After cooling the sample vial, 2 ml n-hexane and 1 ml saturated sodium chloride (NaCl) solution were added, and let stand for 10 min to stratify. The supernatant (hexane fraction) was then transferred for GC-MS analysis.

GC-MS analysis was performed on Agilent 7890 A/5975 C, with specific parameter settings modified according to the previously reported procedures. The samples were separated by VF-23 ms chromatographic column (Agilent: 30 m(length), 0.25 mm (inner diameter), 0.25 μm (film thickness)). FFA composition was determined based on the retention time of validated fatty acid methyl ester standard (Supelco 37, sigma). FFA content was expressed as a percentage of peak area.

### RNA Extraction, Reverse Transcription, and Real-Time Quantitative PCR

The detected 12 genes involved in liver fatty acid metabolism (synthesis, desaturation, uptake, transport and oxidation) were as follows: acetyl-CoA carboxylase 1 (ACC1), fatty acid synthase (FAS), stearoyl‐CoA desaturase 1 (SCD1), delta-5 desaturases (FADS1), delta-6 desaturases (FADS2), CPT1α, Mitofusin1, fatty acid transposase (FAT/CD36), fatty acid transport protein2/4/5 (FATP2/4/5), and liver fatty acid binding protein (L-FABP).

Total RNA was extracted from frozen liver tissue with TRIzol reagent (Invitrogen) and reverse-transcribed using PrimeScript™ RT reagent kit (Takara BIO Inc., Code No. RR047A) according to manufacturer’s instructions. Real-time Quantitative PCR (RT-qPCR) was performed using TB Green^®^ Premix Ex Taq™ II (Tli RNaseH Plus) (Takara BIO Inc., Code No. RR820A) and LightCycler^®^ 96 system. The total volume of RT-qPCR reaction was 10 µl, and its cycling parameters were set as follows: pre-denaturation for 30 s at 95°C, followed by 40 cycles at 95°C for 5 s and 60°C for 60 s, then annealing and elongation. The expression level of each gene relative to GAPDH was determined by the 2-ΔΔCt method, and then normalized to the corresponding control group. Gene-specific primers of hamsters for RT-qPCR are listed in **Table 2**.

**Table 2:** Hamster primer sequences used in this study.

Gene	Forward primer	Reverse primer
ACC1	5′‐TCAAGTCCTTCCTGCTCACACA‐3′	5′‐TCCACCATCACTCAGCCGAT‐3′
FAS	5′‐TTAGCTCTAGTCCCACCCGGAA‐3′	5′‐CACTAGACTCCAGCAGATTAACCC‐3′
SCD1	5′‐GGTACTACAAGCCCGCCAT‐3′	5′‐AGCACCAAAGTGTATCGCAAG‐3′
FADS1	5′‐ACCTCTTTTAATCAGTCCCCAA‐3′	5′‐GCTATACAATGCTGGAACACA‐3′
FADS2	5′‐GCACCTCAACTTCCAGATCGAG‐3′	5′‐CAGGGAACTCACAATGTCCAGCAG‐3′
CPT1α	5′‐GGTTTGACAAGTCCCTCACGTT‐3′	5′‐TCTCCTTTACAATGCCCGTCCT‐3′
Mitofusin1	5′‐TCTTAACAACAAAGGCTGCTCT‐3′	5′‐TCATTACCACAGTCTCGGCAAG‐3′
CD36	5′‐CAAATGCAAAGAAGGAAAGCCTGT‐3′	5′‐GGCTCCACATCCAAGTATGTCC‐3′
FATP2	5′‐TTAAACACCGCAAAGTGACCCT‐3′	5′‐TCACCGGGATACTCAGAGCTT‐3′
FATP4	5′‐GGCAATCAATCTGGACCGACT‐3′	5′‐ACACAAAAGACAGGATTCGGCTA‐3′
FATP5	5′‐CACACCTCATTTCATCCGCATC‐3′	5′‐GTCATAGCTTCCACGTTCCCTC‐3′
L-FABP	5′‐ATCAGAAATCGAGCATAACGGGAA‐3′	5′‐CATCTTAACCACAGCCTTGACC‐3′
GAPDH	5′‐TTGCTGCCATCAATGACCCCTT‐3′	5′‐TTCTCAGCCTTGACTGTGCCTT‐3′

### Western Blotting Analysis

Proteins extracted from hamster liver samples (~ 0.02 g) were separated by 10% or 15% SDS-PAGE, and then transferred to nitrocellulose filter (NC) membranes. The NC membranes were further blocked in 5% skim milk and incubated with primary antibodies of anti-CPT1α (1:1000, 15184-1-AP), anti-L-FABP (1:2000, 13626-1-AP), anti-Mitofusin1 (1:500, 13798-1-AP), and anti-GAPDH (1:3000, 10494-1-AP) overnight at 4°C. The secondary antibody was HRP goat anti-rabbit IgG (1:6000, SA00001-2). Densitometric analysis of WB blots was performed with quantity one software.

### Mitochondria Stain

The fixed liver tissue was embedded in a paraffin block and cut into 4-μm-thick sections. Heat shock protein 60 (HSP60, GB11243, 1:500), a mitochondrial chaperonin, was incubated with sections at 4°C overnight to label the mitochondria. CY3 goat anti-rabbit (GB21303,1:300) was used as the secondary antibody and incubated for 50 min. After washing three times with PBC, nuclei were stained with DAPI for 10 min. Finally, the morphology of mitochondria was observed by fluorescence microscope (NIKON ECLIPSE C1).

### ROS Determination

The liver tissue was prepared into frozen sections (10 μm thick) and then incubated with ROS dye for 30 min. After washing with PBC for three times, nuclei were stained with DAPI for 10 min. Fluorescence was detected in a positive fluorescence microscope (NIKON ECLIPSE C1).

### Statistical Analysis

Statistical comparisons of the data were performed by SPSS 18.0 software using one‐way analysis of variance (ANOVA). The experimental results were presented as mean ± standard deviation (SD). P values < 0.05 were considered statistically significant.

## Results

### Quantitative Changes of FFAs in Hamster Liver Treated With ADT

Quantitative changes of 13 FFAs detected by GC-MS in hamster liver in different experimental groups are shown in **Table 3** (n = 9). These data indicated that total SFA and monounsaturated fatty acid (MUFA) increased significantly in the HFD group compared to the control group. However, there was no obvious change in total Polyunsaturated fatty acid (PUFA) between the two groups, with a slight increase in n-6 fatty acids and a decrease in n-3 fatty acids. ADT treatment significantly reduced total SFA and MUFA in the liver of hamsters fed a high-fat diet, and evidently up-regulated n-3 PUFA.

**Table 3:** Quantitative changes of liver FFAs in hamsters treated with ADT (*n* = 9).

Fatty acids	Control	HFD	HFD+ADT5	HFD+ADT10	HFD+ADT20	HFD+ADT40
(mol/ml)			5 mg/kg	10 mg/kg	20 mg/kg	40 mg/kg
C14: 0 (myristic acid)	0.29 ± 0.10	0.41 ± 0.20	0.32 ± 0.09	0.30 ± 0.06	0.32 ± 0.07	0.28 ± 0.06
C16:0 (PA, palmitic acid)	26.19 ± 4.93	34.40 ± 6.67^###^	26.57 ± 4.20^***^	22.62 ± 2.72^***^	27.42 ± 2.60^**^	27.43 ± 2.73^**^
C18:0 (SA, stearic acid)	16.72 ± 3.05	20.28 ± 3.24^##^	19.89 ± 1.38	16.60 ± 1.91^**^	19.88 ± 1.91	21.31 ± 2.68
Total SFA	43.20 ± 6.80	55.09 ± 8.79^###^	46.78 ± 5.47^**^	39.52 ± 4.53^***^	47.61 ± 4.07^*^	49.03 ± 4.43^*^
C16:1 (palmitoleic acid)	1.51 ± 0.70	3.49 ± 0.92^###^	2.70 ± 0.65^*^	2.33 ± 0.59^***^	2.31 ± 0.46^***^	2.04 ± 0.28^***^
C18:1n9 (oleic acid)	18.76 ± 4.81	52.32 ± 9.46^###^	37.92 ± 5.10^***^	32.98 ± 5.01^***^	34.99 ± 4.65^***^	35.51 ± 4.52^***^
C20:1 (eicosenoic acid)	0.09 ± 0.04	0.27 ± 0.11^###^	0.21 ± 0.04^*^	0.20 ± 0.05^*^	0.20 ± 0.05^*^	0.23 ± 0.08
C24:1 (nervonic acid)	0.25 ± 0.09	0.21 ± 0.04	0.31 ± 0.13	0.26 ± 0.04	0.31 ± 0.15	0.38 ± 0.14
Total MUFA	20.60 ± 5.46	56.29 ± 10.35^###^	41.14 ± 5.72^***^	35.77 ± 5.55^***^	37.82 ± 5.04^***^	38.16 ± 4.71^***^
C18:2n6 (LA, linolenic acid)	21.15 ± 6.76	26.70 ± 4.83^##^	24.17 ± 4.01	20.25 ± 2.85^**^	24.04 ± 3.16	24.70 ± 2.14
C20: 2n6 (eicosadienoic acid)	0.13 ± 0.03	0.21 ± 0.05^##^	0.19 ± 0.03	0.20 ± 0.04	0.20 ± 0.04	0.26 ± 0.08^*^
C20: 3n6 (DGLA, dihomogamma linolenic acid)	0.73 ± 0.15	0.57 ± 0.19	0.75 ± 0.08	0.69 ± 0.08	0.84 ± 0.18	0.94 ± 017^**^
C20:4n6 (AA, arachidonic acid)	7.93 ± 1.32	7.73 ± 1.22	8.94 ± 1.14^*^	8.41 ± 1.43	9.18 ± 0.98^*^	9.66 ± 1.28^**^
Total n-6 PUFA	29.94 ± 7.93	35.21 ± 4.42^#^	34.05 ± 4.97	29.55 ± 2.99^*^	34.26 ± 3.94	35.55 ± 2.91
C18:3n3 (ALA, α-linolenic acid)	0.25 ± 0.17	0.34 ± 025	0.25 ± 0.10	0.20 ± 0.06	0.20 ± 0.07	0.18 ± 0.04
C22:6n3 (DHA, docosahexaenoic acid)	4.52 ± 0.90	3.46 ± 0.45^##^	4.32 ± 0.59^**^	4.17 ± 0.69^*^	4.37 ± 0.59^**^	4.76 ± 0.70^***^
Total n-3 PUFA	4.77 ± 0.97	3.80 ± 0.38^##^	4.57 ± 0.65^*^	4.37 ± 0.70	4.57 ± 0.58^*^	4.95 ± 0.67^**^
Total PUFA	34.71 ± 8.59	39.01 ± 4.30	38.62 ± 5.53	33.92 ± 3.25	38.84 ± 4.37	40.50 ± 3.29

Results are presented as mean ± SD (n = 9). SFA correspond to 14:0, 16:0 and 18:0. MUFA correspond to 16:1, 18:1n9, 20:1, and 24:1. n-6 PUFA include 18:2n6, 20:2n6, 20:3n6, and 20:4n6; n-3 PUFA include 18:3n3 and 22:6n3; PUFA correspond to n-6 and n-3 PUFA. Differences between groups were assessed by one-way analysis of variance (ANOVA) for multiple comparisons followed by LSD or Dunnett’s t-test for post-hoc tests. ^#^p < 0.05, ^##^p < 0.01, and ^###^p < 0.001 versus control. ^*^p < 0.05, ^**^p < 0.01, and ^***^p < 0.001 versus HFD.

The specific changes of different FFAs between the experimental groups were further analyzed. C16:0 as well as C18:0, the two main SFAs, were increased in HFD, and this increase was subsequently inhibited by ADT treatment. C18:1n9, the main MUFA in the body, was significantly elevated in HFD, with concentrations nearly tripling compared to control levels, which can be reduced by ADT administration. For these three FFAs, ADT at 10 mg/kg was the most effective. In addition, C16:1 was also obviously up‐regulated in HFD and was reduced in a dose-dependent manner by ADT. Meanwhile, the essential fatty acids (EFAs) including C18:2n6 and C18:3n3 were increased in HFD group and decreased in HFD + ADT group. In contrast, HFD-fed hamster livers showed lower PUFAs levels, including C20:3n6, C20:4n6, and C22:6n3, which were markedly elevated after ADT administration. Other FFAs (C14:0, C20:1, C20:2, and C24:1) had very low liver concentrations, C14:0 and C24:1 did not differ among the groups, while C20:1 and C20:2 were evidently altered.

### Effects of ADT on Synthesis, Desaturation, and Uptake of Fatty Acids in Liver

**Figure 2:**
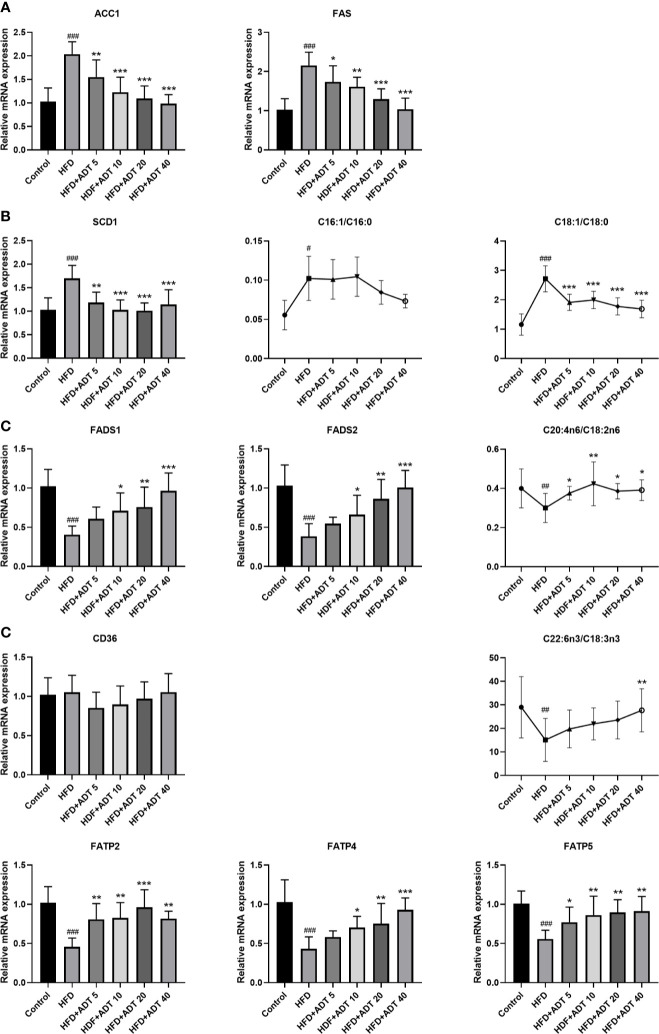
Effects of ADT on FAs synthesis, desaturation and uptake in the liver of hamsters fed high-fat diets (n = 6). **(A)** mRNA levels of ACC1 and FAS. **(B)** mRNA level and activity of SCD1. SCD1 converts SFA into MUFA, so the activity of SCD1 is represented by MUFA/SFA (C16:1/C16:0 and C18:1/C18:0). **(C)** mRNA levels and activity of FADS1 and FADS2. FADS1 and FADS2 metabolize EFAs(LA and ALA) into more unsaturated long-chain PUFAs, so C20:4n6/C18:2n6 and C22:6n3/C18:3n3 are used to represent activity. **(D)** mRNA levels of FAs uptake genes (CD36, FATP2, FATP4, FATP5). Data are presented as mean ± *SD*. Differences between groups were assessed by one-way analysis of variance (ANOVA) for multiple comparisons followed by LSD or Dunnett’s t-test for post-hoc tests. ^#^ *p* < 0.05, ^##^ *p* < 0.01, and ^###^ *p* < 0.001 versus control. ^*^ *p* < 0.05, ^**^ *p* < 0.01, and ^***^ *p* < 0.001 versus HFD.

To investigate the molecular mechanism of ADT, we further quantitatively measured mRNA expression levels of genes related to FA synthesis, desaturation, and uptake in the liver (**Figure 2**). The expression of ACC1 and FAS was significantly increased in the HFD group and decreased dose-dependently in HFD + ADT groups (**Figure 2A**).

SCD1, a key rate-limiting enzyme that desaturates SFA into MUFA, was significantly elevated in the HFD group and attenuated by ADT administration. The changes of SCD1 were associated with changes in C16:1/C16:0 and C18:1n9/C18:0 ratios (**Figure 2B**). On the contrary, FADS1 and FADS2, key rate-limiting enzymes in the desaturation of PUFA, were obviously down-regulated in HFD and dose-dependently increased by ADT treatment. Similarly, the changes in FADS1 and FADS2 were consistent with changes in C20:4n6/C18:2n6 and C22:6n3/C18:3n3 ratio (**Figure 2C**).

Moreover, genes involved in liver FA uptake showed that the expression of CD36 was not obviously different among the groups, while the expression of FATP2, FATP4, and FATP5 was significantly reduced in HFD group but activated by ADT therapy (**Figure 2D**).

### Effects of ADT on FAs Transport, β-Oxidation, and Mitochondrial Fusion in Liver

**Figure 3:**
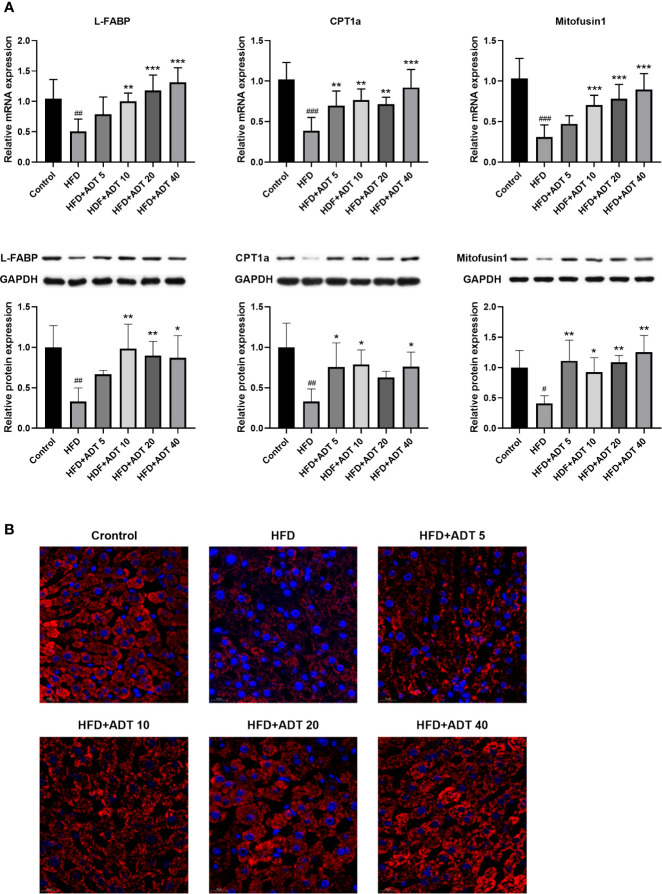
Effects of ADT on FAs transport, β-oxidation, and mitochondrial fusion in the liver of hamsters fed with HFD. **(A)**: mRNA and protein levels of L-FABP, CPT1α, and Mitofusin1 (n = 6). CPT1α catalyzes FAs into mitochondria; Mitofusin1 regulates the fusion state of mitochondria. Data are presented as mean ± *SD*. Differences between groups were assessed by one-way analysis of variance (ANOVA) for multiple comparisons followed by LSD or Dunnett’s t-test for post-hoc tests. ^#^ *p* < 0.05, ^##^ *p* < 0.01, and ^###^ *p* < 0.001 versus control. ^*^ *p* < 0.05, ^**^ *p* < 0.01, and ^***^ *p* < 0.001 versus HFD. **(B)** Fluorescent images of mitochondria labeled with HSP60 in hamster liver (magnification, ×600).

Excessive free FAs in hepatocytes are bound/isolated by L-FABP and then transported to various cell compartments for rapid removal, mainly by mitochondrial β-oxidation. Therefore, the mRNA and protein expression levels of L-FABP, CPT1 and Mitofusin1 were quantitatively measured, and the liver mitochondria were stained to observe their fusion status (**Figure 3**). As shown in **Figure 3A**, L-FABP, CPT1α, and Mitofusin1 were significantly decreased in HFD group, but remarkably raised in the HFD + ADT group. Furthermore, ADT treatment strikingly promoted mitochondrial fusion in HFD-fed hamster hepatocytes, which was consistent with the expression changes of Mitofusin1 (**Figure 3B**).

### Effect of ADT on ROS and Liver Function of HFD-Fed Hamsters

**Figure 4:**
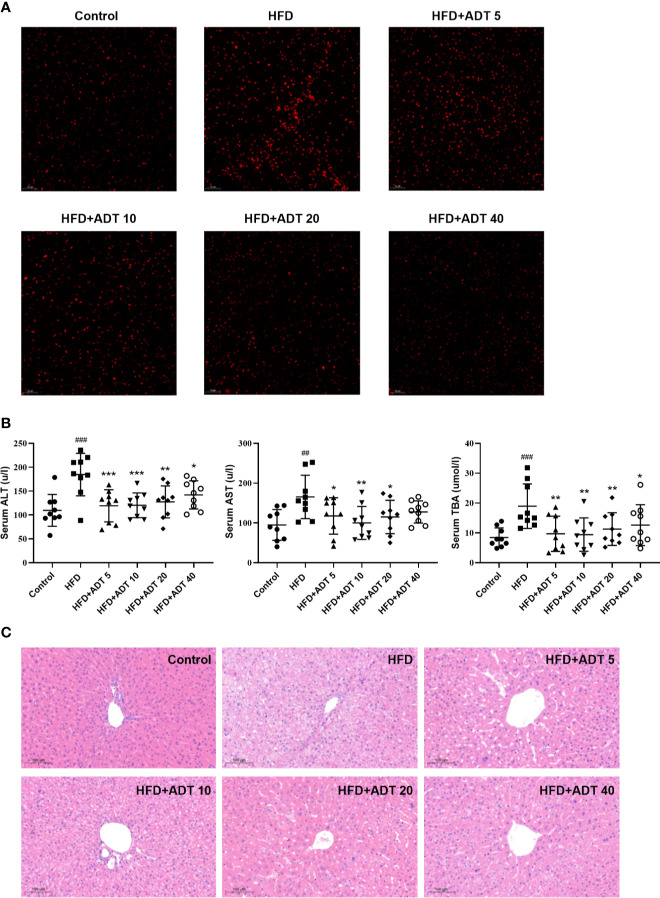
Effect of ADT on ROS and liver function of HFD-fed hamsters. **(A)** Fluorescent images of ROS in hamster liver (magnification, ×200). **(B)** Serum levels of ALT, AST, and TBA in each group of hamsters (n = 9). ALT, alanine aminotransferase; AST, aspartate aminotransferase; TBA, total biliary acid. Data are presented as mean ± *SD*. Differences between groups were assessed by one-way analysis of variance (ANOVA) for multiple comparisons followed by LSD or Dunnett’s t-test for *post hoc* tests. ^##^ *p* < 0.01, and ^###^ *p* < 0.001 versus control. ^*^ *p* < 0.05, ^**^ *p* < 0.01, and ^***^ *p* < 0.001 versus HFD. **(C)** Histopathological examination of hamster liver sections by HE staining (magnification, ×100).

To establish the major defect of abnormal FAs metabolism driving by HFD, we performed fluorescence staining of liver ROS. The results showed that HFD significantly increased the level of ROS, while ADT administration dramatically reduced the excessive accumulation of ROS (**Figure 4A**).

Serum ALT, AST, and TBA levels were measured to assess the effect of ADT on liver function. As shown in **Figure 4B**, different doses of ADT significantly reduced elevated ALT, AST, and TBA levels in hamsters fed a high-fat diet. In addition, changes in these biochemical indicators have been confirmed by pathological changes in the liver. H&E staining results revealed that significant swelling of hepatocytes and increased lipid droplets in the liver of HFD-fed hamsters, while control hamsters displayed normal liver histology. Compared with the HFD group, liver steatosis in ADT intervention groups with different concentrations were improved to varying degrees, and liver structure tended to normal (**Figure 4C**).

## Discussion

FFA-induced lipotoxicity plays a pathological role in the occurrence and development of many metabolic diseases, however, current therapeutic strategies are limited. Our results indicated that ADT administration significantly reduced the concentration of toxic SFA and MUFA in HFD hamster liver, and increased the level of n-3 PUFA. In addition, ADT obviously decreased serum ALT, AST, TBA and liver ROS levels, and improved hepatic steatosis. These results confirmed that ADT can effectively improve FAs metabolic disorders and Liver damages caused by HFD, making ADT a promising candidate drug for lipotoxicity therapy.

Most data indicate that lipotoxicity mainly originates from toxic SFAs, in particular palmitic acid (PA, C16:0) and stearic acid (SA, C18:0) (Win et al., 2015; Lu et al., 2016). PA and SA overload induces cell dysfunction or death through multiple mechanisms, including increased synthesis of harmful compound lipids (diacylglycerol and ceramide), endoplasmic reticulum (ER) stress, impaired mitochondrial function, toll-like receptor 4 (TLR4) -mediated inflammation, and activation of death receptors (Chavez and Summers, 2003; Anderson et al., 2012; Salvado et al., 2015; Win et al., 2015). In this study, HFD-fed hamster livers showed increased level of SFAs, especially PA, while ADT treatment significantly inhibited this overaccumulation. This may partially explain the protective effect of ADT on liver injury, and we further explored the molecular mechanisms underlying these changes.

Hepatic SFAs are mainly derived from *de novo* lipogenesis (DNL), adipose tissue and diets. Under physiological conditions, free FAs are bound/sequestered by L-FABP and then transported to oxidative (peroxisomes, mitochondria) or stored (endoplasmic reticulum, lipid droplets) organelles for rapid removal, minimizing the toxic effects of excess free FAs (Smathers et al., 2013; Wang et al., 2015). Mitochondrial β-oxidation is the most important metabolic pathway of FAs. Of note, Rambold et al. recently found that in nutrient-deficient Mitofusin1 knockdown MEFs, only fused mitochondria can efficiently take up FAs and ensure its homogenous distribution throughout the mitochondria, maximizing the use of FAs for β-oxidative reactions (Rambold et al., 2015). Therefore, this study investigated the expression of genes involved in FAs metabolism, including synthesis, oxidation, uptake, and transport. We found that HFD induced overexpression of ACC1 and FAS, while the levels of L-FABP, CPT1α, and Mitofusin1 were significantly down-regulated, and mitochondria were also fragmented. Increased synthesis, weakened binding/transportation, and impaired β-oxidation are concurrently operative to provide a continuous excess of ROS and free FAs, especially PA. These excess PA and its toxic metabolites can activate various intracellular reactions, leading to lipotoxic stress in the endoplasmic reticulum and mitochondria, which in turn exacerbated the accumulation of free FAs. Compared with HFD group, ADT administration significantly inhibited ACC1 and FAS, up-regulated L-FABP, CPT1α, and Mitofusin1, and promoted mitochondrial fusion, thereby effectively reducing the accumulation of toxic SFAs and improving liver damage.

Appropriate intracellular balance of SFAs and MUFAs is regulated by SCD1 (Singh et al., 2015). SCD1 catalyzes the desaturation of SFA to MUFA, mainly producing oleic acid (OA, C18:1n9). It has been reported that OA is mainly incorporated into relatively inert triacylglycerol (TAG) and stored in lipid droplets, so metabolizing SFA to OA *via* SCD1 reduces the excessive formation of toxic lipid intermediates (diacylglycerol and ceramides) (Man et al., 2006; Palomer et al., 2018). In this study, long-term HFD induced SCD1 and significantly increased the concentration of OA, which may be due to the body’s adaptive results in the face of on-going metabolic stress. However, this relative protection by channeling the SFA into the less toxic lipid pool is only a temporary measure, as the TG buffer capacity in liver tissue is limited. Continuous overproduction of OA results in liver lipid accumulation, steatosis, and low-grade chronic inflammation, thereby potentiating the metabolic syndrome (Ricchi et al., 2009; Singh et al., 2015). In addition to effectively reducing the accumulation of toxic SFA, our results indicated that ADT treatment also inhibited SCD1 overexpression and significantly decreased the content of MUFA (mainly OA).

Unlike SFAs and MUFAs, PUFAs have important physiological functions in the body and are associated with beneficial health effects. Emerging evidence shows that excessive intake of HFD reduces the levels of AA (C20:4n6), EPA (C20:5n3), and DHA (C22:6n3), enhances the production of pro-inflammatory arachidic acids and reactive oxygen species (ROS), leading to pro-inflammatory status (Das, 2018; Gai et al., 2018). AA, EPA and DHA are endogenously derived from essential fatty acids (EFAs: LA, C18:2n6; ALA, C18:3n3) through a successive series of desaturation and chain extension steps. The rate-regulating conversion steps in this process are mediated by Δ-6 desaturase (FADS2) and Δ-5 desaturase (FADS1) (Koletzko et al., 2019). Our results also confirmed that HFD impaired the desaturated metabolic pathway of EFAs: the ratio of AA/LA and DHA/ALA in HFD hamsters was down-regulated, and the expressions of FADS1 and FADS2 were significantly decreased. In this study, the liver concentration of EPA was too low for accurate quantification. ADT administration obviously up-regulated FADS1 and FADS2, increasing the conversion of EFA to AA and DHA.

**Figure 5:**
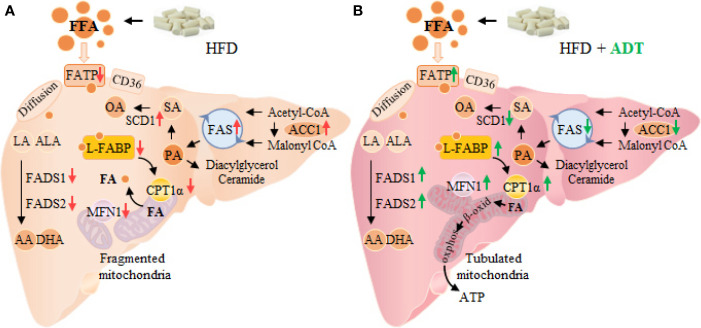
Changes of FAs metabolism in hamster liver of HFD group **(A)** and HFD+ADT group **(B)**. Compared with the HFD group, ADT administration significantly inhibited the overexpression of ACC1, FAS and SCD1, activated FADS1 and FADS2, thereby reducing endogenous synthesis of PA, SA and OA, and improved the desaturated metabolic pathway of EFAs. In addition, ADT upregulated the levels of FATPs, L-FABP, and CPT1α, thus increasing the uptake, binding/isolation, transport, and β-oxidation of FAs. More importantly, ADT obviously increased the expression of Mitofusin1 to promote mitochondrial fusion state and maximal β-oxidation. PA, palmitic acid; SA, stearic acid; OA, oleic acid; EFAs, essential fatty acids, including LA and ALA; MFN1, Mitofusin1.

In conclusion, this study demonstrates that ADT is effective in improving HFD induced fatty acid metabolism disorders and liver injuries. Specifically, ADT may exert protective effects by: (i) inhibiting overexpression of ACC1, FAS, and SCD1, thereby reducing endogenous synthesis of toxic palmic acid (C16:0) and oleic acid (C18:1); (ii) up-regulating FATPs, L-FABP, CPT1α, and Mitofusin1, thus enhancing the β-oxidation of liver fatty acids; (iii) activating FADS1 and FADS2 to improve the desaturated metabolic pathway of EFAs (outlined in **Figure 5**). These results may provide important new insights into the role of ADT in hepatic FAs metabolism and perhaps new therapeutic strategies for lipotoxicity. However, further researches are still needed to evaluate the potential application of ADT in the clinical treatment of lipotoxicity-induced metabolic diseases (eg, NAFLD).

Moreover, there is one further point to make. The clinically available H_2_S sustained-release donor ADT is a prodrug, and it is difficult to determine whether its regulatory effect on liver FFAs depends on itself or H_2_S. In our opinion, H_2_S largely mediated the FFAs regulation of ADT. First, ADT is rapidly demethylated into ADT-OH *in vivo*, and their dithiolethione pharmacophore is cleaved by liver microsomes to release H_2_S (Wang et al., 2014; Szabo and Papapetropoulos, 2017). Second, more and more evidences show that H_2_S mediates the biological effects of ADT and ADT-OH. For example, Zhang et al. found that ADT and ADT-OH can activate Nrf2 signaling through the S-sulfhydration of Keap1 (Zhang and Munday, 2008). In 2013, Yang et al. used NaHS and CSE knockout mice (lacking H2S production), indicating that H2S also activated Nrf2 by S-sulfhydration of Keap1 (Yang et al., 2013). Besides, both ADT-OH and NaHS can enhance AMPK activation in brain regions where microglia cells are over-activated under LPS stimulation (Zhou et al., 2014). Therefore, these results support that H_2_S released from ADT or ADT-OH mediates its biological effects, but further studies are needed.

## Data Availability Statement

The raw data supporting the conclusions of this article will be made available by the authors, without undue reservation.

## Ethics Statement

The animal study was reviewed and approved by the Laboratory Animal Care and Welfare Committee of Central South University.

## Author Contributions

YL and CZ contributed to the conception and design of this study. CZ and NY conducted the experiments. MT, HC, WL, NY, and CZ contributed to the detection of liver free FAs and related gene levels. CZ and WL performed data processing and statistical analysis. CZ wrote the first draft of the manuscript. All authors contributed to the article and approved the submitted version.

## Conflict of Interest

The authors declare that the research was conducted in the absence of any commercial or financial relationships that could be construed as a potential conflict of interest.

## Abbreviations

ADT, anethole dithiolethione, 5-(4-methoxyphenyl)-3H-1,2-dithiole-3-thione; ACC1, acetyl-CoA carboxylase 1; CPT1α, carnitine palmitoyl-transferase 1α; EFAs, essential fatty acids; FAs, fatty acids; FAS, fatty acid synthase; FADS1, delta-5 desaturases; FADS2, delta-6 desaturases; FAT/CD36, fatty acid transposase; FATP, fatty acid transport protein; H_2_S, hydrogen sulfide; HFD, high-fat diet; L-FABP, liver fatty acid binding protein; Mitofusin1, mitochondrial fusion protein 1; MUFAs, monounsaturated fatty acids; NAFLD, non-alcoholic fatty liver disease; PUFAs, polyunsaturated fatty acids; SFAs, saturated fatty acids; SCD1, stearoyl-CoA desaturase 1.
